# Lying-in Hospital at Vienna

**Published:** 1852-02

**Authors:** 


*Lying-in Hospital at Vienna.*—In the Lying-in Hospital, inviolable secresy is observed, if required. The female may enter masked, accomplish her delivery, and depart when able, without her name being known, or even her face seen. This is the case where the patient is illegitimately with child, or if married, is able to pay. If *married* and poor, she can only be admitted on certificate of her poverty, and then her expenses are assessed upon her relatives or parish.

As a necessary result from this premium on immorality, the proportion of illegitimate to legitimate births in the city of Vienna, has been found to be as one to two and a quarter; a proportion never equalled, except in the city of Munich, where, in the year 1838, the number of illegitimates *exceeded* the legitimate births by 270. Another result is a frightful mortality among the foundlings, amounting in 1784 to 54 per cent., and in 1812 to 69 per cent. of the number received.

In thus sanctioning such fearful social evils, the Austrian government have outraged all that we are accustomed to regard as most sacred in humanity; but the world has ceased to expect either liberality, or honesty, or philanthropy, or honor, from a government notorious for the opposite qualities.—*New York Medical Gazette.*

